# Twelve protections evolved for the brain, and their roles in extending its functional life

**DOI:** 10.3389/fnana.2023.1280275

**Published:** 2023-11-06

**Authors:** Jonathan Stone, John Mitrofanis, Daniel M. Johnstone, Stephen R. Robinson

**Affiliations:** ^1^Faculty of Medicine and Health, University of Sydney, Camperdown, NSW, Australia; ^2^Grenoble and Institute of Ophthalmology, Fonds de Dotation Clinatec, Université Grenoble Alpes, University College London, London, United Kingdom; ^3^School of Biomedical Sciences and Pharmacy, University of Newcastle and School of Medical Sciences, The University of Sydney, Camperdown, NSW, Australia; ^4^School of Health and Biomedical Sciences, RMIT University, Bundoora, VIC, Australia; ^5^Institute for Breathing and Sleep, Austin Health, Heidelberg, VIC, Australia

**Keywords:** brain evolution, dementia, brain protection, acquired resilience, intracerebral haemorrhage, neurotoxicity

## Abstract

As human longevity has increased, we have come to understand the ability of the brain to function into advanced age, but also its vulnerability with age, apparent in the age-related dementias. Against that background of success and vulnerability, this essay reviews how the brain is protected by (by our count) 12 mechanisms, including: the cranium, a bony helmet; the hydraulic support given by the cerebrospinal fluid; the strategically located carotid body and sinus, which provide input to reflexes that protect the brain from blood-gas imbalance and extremes of blood pressure; the blood brain barrier, an essential sealing of cerebral vessels; the secretion of molecules such as haemopexin and (we argue) the peptide Aβ to detoxify haemoglobin, at sites of a bleed; autoregulation of the capillary bed, which stabilises metabolites in extracellular fluid; fuel storage in the brain, as glycogen; oxygen storage, in the haemoprotein neuroglobin; the generation of new neurones, in the adult, to replace cells lost; acquired resilience, the stress-induced strengthening of cell membranes and energy production found in all body tissues; and cognitive reserve, the ability of the brain to maintain function despite damage. Of these 12 protections, we identify 5 as unique to the brain, 3 as protections shared with all body tissues, and another 4 as protections shared with other tissues but specialised for the brain. These protections are a measure of the brain’s vulnerability, of its need for protection. They have evolved, we argue, to maintain cognitive function, the ability of the brain to function despite damage that accumulates during life. Several can be tools in the hands of the individual, and of the medical health professional, for the lifelong care of our brains.

## Introduction—not taking the brain for granted

The human brain needs no introduction, not its huge size, nor its complexity, nor its centrality in our health and psychology. We take it for granted in our childhood but soon enough learn, from observing dementia in our grandparents’ generation, then in our parents’, the tragedy of a failing brain. And then, we wonder about ourselves. This essay explores understanding of the brain’s durability and vulnerability in evolutionary terms, seeking insights to guide the understanding of this awful aspect of human ageing, that dementia is our fate—if we survive the slings and arrows of youth and mid-age—sealed by the realities of vascular ageing (Stone et al., 2015; Andersson and Stone, 2023). Briefly, the insights are three.

First, there has been a huge “evolutionary investment” in the protection of the brain, but these mechanisms are also a measure of how vulnerable the brain is; else how could they have evolved? Second, one of the mechanisms of protection, acquired resilience (Stone et al., 2018), provides the conceptual basis for organising life-long defence of the brain. Third, the analysis draws attention to the brain’s remarkable cognitive reserve—its ability to continue full cognitive function, even while sustaining damage—until that reserve is exhausted, and we start to forget what once we remembered. Protection of that cognitive reserve has the potential to extend our full cognitive life from the seventy “days of our years” recognised in the scriptures,^1^ for another decade or two or more. It is something to fight for.

## Five protections unique to the brain

### Protection from piercing or crushing: the cranium

The cranium is the most complex of the body’s bones, comprising eight or more hard bones (arguably 16 or more, since they come in pairs). Its detail has been known for centuries, but the evolutionary “investment” in its complexity is commented on only rarely. There is further “investment” in its developmental pattern. It remains flexible through birth; its bones become fused together in early childhood, well before the capping of growth of the long bones of the limbs; yet growth continues at the sutures, somehow matching the growth of the brain. The cranial bones form an effective helmet, blocking piercing injuries and spreading the pressure of a knock over the whole side of the brain.

We note one complication created by this bony protection. If the brain swells in disease, or after injury, or if an artery bleeds into the brain, then because the cranium is so rigid, intracranial pressure can rise and can squeeze the brain outwards through the foramen magnum. The process is called “coning” or “herniation” (Tadevosyan and Kornbluth, 2021) and can be quickly fatal; the emergency options include rapid removal of a patch of the cranium, allowing a more controlled, less lethal extrusion of part of one of the forebrain lobes. At such moments, a more stretchable cranium would be an advantage.

### Protection from rocking and rolling: suspension in the cerebrospinal fluid

The living brain is soft and gel-like, and quite heavy—1.2–1.4 kg in young adults (Hartmann et al., 1994). The evolved mechanism to minimise the movement of the brain within the cranium is for the brain to float in cerebrospinal fluid (CSF), which floods a narrow gap between the surface of the brain and the cranial bones. Floating in CSF, the brain’s apparent weight is ~50 g and its movement within the cranium is greatly reduced. When emergency surgeons dealing with a fractured cranium note leakage of CSF, often from the nose or ear, they give close attention and may intervene surgically (Phang et al., 2016), until the “float” of the brain can be re-established.

The complexity of this hydraulic support includes specialisations of the dura mater to form stiff folds between the two hemispheres of the forebrain (the falx cerebri) and between the cerebrum and cerebellum (the tentorium cerebelli). These folds are considered to act as baffles within the cranial vault, further stabilising the brain. In addition, there is a slow circulation of CSF from where it is generated, from the ependyma and choroid plexuses inside the brain, through consistent foramina in the pia mater into the subarachnoid space, and then through arachnoid granulations into the dural venous sinuses. Because of the complexity of these structures that float the brain, failures do occur, as in hydrocephalus (Kahle et al., 2016), evident in infants as macrocephaly (an enlarged head, because raised intracerebral pressure inflates the skull); or, if it occurs after the skull’s sutures have sealed, as persistent headaches and often enlarged lateral ventricles, requiring surgical intervention.

### Protection from blood gas imbalance: the carotid body reflex

The common carotid arteries arise (left and right) as branches from the aorta (the right via the subclavian artery), to run vertically up into the neck. At the level of the upper end of the larynx, each common carotid bifurcates, one branch (the external carotid) running superficially, to supply tissues of the side of the head, while the other (the internal) ascends to the base of the cranium, through which it passes in a bony canal, to supply the brain. The two internal carotid arteries supply ~75% of the brain; the other 25% is supplied by the two vertebral arteries, which enter the cranium through the foramen magnum.

At the carotid bifurcation, the common carotid artery shows a slight swelling, the carotid sinus, which sometimes extends to include the beginning of the internal carotid. On the rear aspect of the sinus a cluster of specialised nerve cells is found, the carotid body. These strategically placed specialisations (the sinus and body) contain sensors that control heart rate, by a reflex regulation of the heart’s pacemaker. Cells of the carotid body monitor oxygen and carbon dioxide and pH levels in the blood, as well as blood glucose and lactate. Activation of carotid body cells induces compensatory changes in respiration, to stabilise blood gas levels (Lopez-Barneo, 2022).

The adjustments in heart rate and respiration effected by the carotid sinus and body (below) affect all the body; but the location of these sensors at the origin of the major arteries supplying the brain suggests that they evolved specifically to maintain and protect the function of the brain.

### Protection from hypertension: the carotid sinus reflex

The carotid sinus contains receptors that respond to the stretch of the sinus wall, activating a baroreceptor reflex, which slows the heart; the output arm of the reflex is transmitted by fibres in the glossopharyngeal nerve (Andani and Khan, 2023), whose activity slows the heart’s pacemaker. The effect is that a sudden rise in blood pressure slows the heart, to protect the brain from hypertension. Conversely, if blood pressure falls, as when we stand, the same reflex, with opposite polarity, induces a rise in heart rate, to maintain blood flow to the brain, and prevent dizziness or loss of consciousness (Andani and Khan, 2023). One clinical complication of the reflex is that its hyperactivity, occurring for reasons often unknown, can cause unconsciousness (syncope), often resulting in falls, particularly in the elderly, because it slows the heart inappropriately and reduces blood supply [reviewed in Amin and Pavri (2015)].

### Protection from excitotoxicity, pathogens and immune cytotoxicity: the blood brain barrier

The blood brain barrier (BBB) is a “highly regulated interface” between two very different tissues (blood, brain) (Galea, 2021). While much is known about the BBB, the question of why it evolved—what survival advantage it provided—is less discussed. One possibility, important to the neurophysiologist, is that the evolution of the CNS as a system using electro-chemical signalling required control of its chemical environment. Thus, the most common CNS transmitter is the glutamate (the ionic form of the amino acid, glutamic acid) and there is a large literature on glutamatergic transmission [reviewed in Smith (2000) and Zhou and Danbolt (2014)], on the production of glutamate by neurones and its recycling by neuroglia, and on its dysfunction, particularly the killing of neurones by excitotoxicity (Zhou and Danbolt, 2014) driven by excess glutamate. As part of this management, which makes glutamate signalling possible, the endothelial cells that form part of the barrier are able actively to transport glutamate out of the brain into the blood (Smith, 2000), against a concentration gradient. A similar “blood-nerve-barrier” has evolved for peripheral nerves and ganglia (Ubogu, 2020), confirming the importance of this function of the BBB for neural signalling. Some investigators (Zhou and Danbolt, 2014) consider the role of the BBB in “shielding” the brain from glutamate in the blood, where its concentration is orders of magnitude higher than the levels toxic to neurones in the brain, as perhaps its major role.

Even so, the primary function of the BBB is often described in immunological terms, as the protection of the brain by preventing the entry of pathogens into it (Galea, 2021). This prevention-of-entry to bacteria and viruses, imposed by the tight junctions between endothelial cells, forms in ontogeny by “instruction” of endothelial cells by astrocytes (and, in the retina, by Müller cells) to form “tight” junctions between each other (Tout et al., 1993). The same barrier properties also prevent entry of the “killer” T-cells of the immune system, which seek out and kill host cells infected by a pathogen, from entering the brain. Dealing with an infection of the brain by killing the infected neurones might kill so many neurones that it would cause a form of dementia, as occurs when the brain is infected, as in syphilis (Mehrabian et al., 2012) or by the HSV-1 (Campos et al., 2021) and HIV-1 viruses (Ances and Ellis, 2007).

In summary, the BBB seems have evolved to establish the conditions for neurotransmission, “shielding” the brain from toxic levels of neurotransmitters like glutamate in the blood, but it seems also to serve two further protective functions, at least: against pathogens and against immune cytotoxicity. The BBB creates at least two complications. Some viruses have evolved an ability to slip through the BBB, and cause infections that too often are fatal because, once in the brain, the viruses are protected from killer T-cells. This problem is exacerbated when there is a cerebral haemorrhage, or just a breakdown of the barrier as a result, for example, of inflammation. Until the barrier is resealed, blood-borne pathogens can enter the brain’s neuropil and infect neurones, creating the conditions for pathogen-induced immune-cytotoxicity, causing the viral dementias mentioned above. A second complication is that the BBB makes difficult the delivery to the brain of most drugs; it has been estimated (Pardridge, 2005) that 98% of “small-molecule” drugs and 100% of “large-molecule” drugs cannot cross the barrier. This makes difficult, for example, the use of chemotherapy for brain tumours (Uluc et al., 2022) and has stimulated the search for ways of facilitating drug access to the brain (Tang et al., 2019; Liu and Jiang, 2022).

## Three protections common to other body tissues

The brain shares the protective benefit of at least three mechanisms that have evolved to protect all body tissues.

### Protection against running out of fuel: glycogen storage

The brain is an energy-hungry organ, requiring “food” in the form of glucose, which the gut extracts from ingested food into the blood, which delivers it to the brain, usually after initial storage in the liver. The glucose is actively transported across the blood brain barrier to reach both neurones and neuroglia, which store some of the glucose reaching them as glycogen, a polymer of glucose, principally in astrocytes. In hypoglycaemia, as in prolonged hunger, the glycogen stored in astrocytes is an on-the-spot source of glucose for neurones and the astrocytes that support them (Falkowska et al., 2015; Prats et al., 2018).

Evidence of the effectiveness of glycogen storage in the brain has come from studies of how glycogen stored in astrocytes is mobilised to provide glucose for neurones (which depend on astrocytes for many forms of support). Glycogen appears to be metabolised in astrocytes to lactate (an anaerobic step, so not requiring oxygen), which is then “shuttled” to neurones, where the lactate becomes fuel in pathways of oxidative phosphorylation, so requiring oxygen and yielding the energy currency of all cells, ATP. If, for example, this movement of lactate from astrocytes to neurones is inhibited experimentally just prior to a “memory consolidation” event, the consolidation is impaired (Dienel, 2019).

The discovery of glycogen storage in tissues is attributed to Claude Bernard, who worked in the middle of the 19th Century (Curtino and Aon, 2019). There is one complication (at least) resulting from this mechanism for fuel storage. The glycogen storage diseases are genetic failures of enzymes that break stored glycogen down to glucose, ready to be metabolised. The resulting malaises are not severe enough to cause death of the embryo or foetus but create significant problems in the individuals who are then born with the enzyme failure. These diseases can be life-shortening, because glycogen continues to be stored, and accumulates damagingly in body tissues (Ozen, 2007). In the brain, for example, Lafora disease is a genetically determined glycogen storage disease that predominantly affects astrocytes and manifests in adolescence as increasingly severe epileptic seizures and a rapid decline in cognitive function (Duran and Guinovart, 2015).

### Protection against running out of oxygen; oxygen storage

The discovery that the brain stores oxygen came in the year 2000, 150 years after the discovery of glycogen storage, with the description of an oxygen-binding haemoprotein in neurones and neuroglia (Burmester et al., 2000). This protein, dubbed neuroglobin, was the third oxygen-carrying haemoprotein described for humans, after haemoglobin (which carries oxygen in the blood) and myoglobin (muscle). A fourth oxygen-carrying haemoprotein, cytoglobin, was described a year later, initially in liver tissue (Yoshizato et al., 2016). The value of neuroglobin as a store of oxygen for the brain has been validated by several observations (Greenberg et al., 2008; Williams et al., 2008; Schneuer et al., 2012), including the upregulation of its expression in nerve cells and astrocytes in conditions of hypoxia, by its high level of expression (several times higher than in land animals) in long-duration-diving sea mammals like the porpoise and the whale, and by evidence that neuroglobin-mediated storage of oxygen reduces damage done to the brain in rodent models of hypoxia. As with the storage of glucose as glycogen, the storage of oxygen bound to neuroglobin is quietly effective; it is easily taken for granted.

Several studies have looked at neuroglobin in the brains of patients with a diagnosis of Alzheimer’s dementia. Sun et al. (2001) provided evidence, subsequently confirmed (Fiocchetti et al., 2017), that neuroglobin is upregulated by hypoxia and acts to mitigate the damage caused to neurones by the hypoxia. They went on to show (Sun et al., 2013) that neuroglobin levels are higher in early- and mid-stage dementia, falling in severe disease. This upregulation gives support to the view that hypoxia occurs early in the pathogenesis of some dementias, caused—many argue—by small haemorrhages (Cullen et al., 2005; Stone, 2008; Stone et al., 2015).

As to complications, there would seem to be two. The possibility has been suggested that the oxygen available from neuroglobin may facilitate the survival of cancers in the brain (Fiocchetti et al., 2017); and, more generally, the evolution of neuroglobin—as of haemoglobin—emphasises that mammals are oxygen-dependent. Without respiration, the brain is damaged within minutes. There is a term for this dependence—we are “obligate aerobes”—and that dependence is quickly and often fatal, even with the oxygen stored in cyto- and neuroglobin.

### Protection from daily stresses, plus the opportunity to extend cell health: acquired resilience

The concept of acquired resilience has been argued in detail previously (Stone et al., 2018). It provides a conceptual and empirical basis for understanding how everyday stresses—hunger, the toxins in dietary plants (Mattson and Cheng, 2006; Stone et al., 2018), solar radiations, hypoxia including the hypoxia of exercise, heat and physical stresses—all can induce tissue resilience. “Resilience” has many phenotypes, often determined by the interests of investigators, including faster wound healing, the conditioning of undamaged tissue against subsequent stress, slowing of age-related degenerations of brain and retina, slowing of age-related degeneration of the skin, slowing of sarcopaenia, reduction of genotoxicity, reduction of inflammation and associated pain, increased mitochondrial production of ATP, supernormal function of retina and muscle and suppression of cancer. Most work has been done on skin, muscle, the central nervous system and the oral mucosa; resilience may be stress-inducible in all tissues. The evidence that acquired resilience can delay, slow and even partially reverse degenerations of the retina and brain has been reviewed (Stone et al., 2018). Many reviews are available for the several stresses that induce tissue resilience—including plant toxins (Mattson and Cheng, 2006), photobiomodulation (Valverde and Mitrofanis, 2022), exercise-induced hypoxia (Valenzuela et al., 2020), and physical stresses such as heat (Laukkanen et al., 2017).

The stresses that induce acquired resilience are hormetic; that is, at high doses they are destructive but, at managed low doses, they induce a resilience response, associated with low morbidity and increased longevity (Mattson and Calabrese, 2010). Some of these relationships are already widely understood as “healthful,” like the benefits of exercise. Acquired resilience provides a conceptual basis for understanding the broad spectrum of stress-induced benefits.

Of the twelve protections in Table 1, acquired resilience offers the best opportunity for the individual to intervene to extend their own brain health. How? By a diet with its share of plant toxins, combined with the hypoxia of exercise, heat as in saunas, weight control, controlled hunger (as in a 5/2 diet) and radiations like photobiomodulation, widely used for wound healing. As to complications, there appear to be few if any, at the effective low doses.

**Table 1:** Twelve mechanisms that protect the brain. Of the 12, 5 are specific to the brain; 3 operate similarly in other body tissues. The remaining 4 operate in all tissues but appear to have become specialised in the brain.

Protective mechanism	Protection against	Problems created
**Mechanisms unique to the brain**		
The cranium—a helmet	External trauma	Brain can be compressed inside
Floating of brain in CSF inside the cranium	Rocking and rolling	Hydrocephalus
Carotid body reflex	Hypoxaemia	—
Carotid sinus reflex	Hypertension and hypotension	Overactivity can cause syncope
Blood-brain barrier	Pathogens and cellular immunity	Blocks delivery of valuable drugs to brain
**Mechanisms shared equally with other organs and tissues**		
Glycogen storage	Lack of fuel (glucose)	Glycogen storage diseases
Oxygen storage	Lack of O_2_, to burn glucose	Possibly, facilitation of cancer
Acquired resilience	Tissue ageing	—
**Mechanisms shared with other organs and tissues but specialised in the brain**		
Cerebral autoregulation	Loss of ionic homeostasis (the specialisation is the pericyte-mediated contractility of capillaries)	—
Generation and wiring of new functional cells in adult	Depletion of neurons (the specialisation is the wiring of neurons into damaged circuitry)	Gliomas, neuromas
Endogenous mechanisms of protection against intracerebral haemorrhage	The toxicity of Hb/haem/haemin/Fe (the specialisation is the brain-specificity of some protectors, including Aβ)	Aβ mistaken as the cause of dementia
Plasticity and rewiring of brain circuitry	Age-cumulative damage to brain circuitry (the specialisation is the rewiring of existing circuitry around damage)	—

## Four protections shared with other tissues but specialised for the brain

### Protection against ionic and metabolic imbalance: autoregulation of cerebral capillaries

Autoregulation is a set of physiological mechanisms (myogenic, neurogenic, signalling) that act, at the local level within organs, to maintain blood flow and metabolite concentrations at physiological levels, over a wide range of blood pressures (Armstead, 2016). The mechanisms of autoregulation have been studied intensively in the brain and retina and are controlled by a “molecular conversation” between neural tissue and its vessels. Perhaps the major outcome of the conversation is that the metabolic state of brain tissue controls the flow of blood through it, at the local level (Iadecola, 2017). This allows for local variations (surges or reductions) in blood flow, separate from the more global control of flow exerted by pulse rate and pulse pressure. Autoregulation adjusts blood flow to function, down to the capillary level.

At the neurovascular interface, between vessels and the neuropil, the processes of both neurones and neuroglial cells (particularly astrocytes) make contact with small blood vessels (arteries, arterioles, capillaries), in patterns that vary with vessel size (Schaeffer and Iadecola, 2021). This interface has been called the “neurovascular unit” (Iadecola, 2017), a concept later included in the “neurovasculome” (Iadecola et al., 2023). Recent reviews of this interface include a tracing of the history of the understanding of its role (Iadecola, 2017; Poole et al., 2020) and reviews of the signals involved (Thakore et al., 2021) and of how pericytes function and malfunction in their constriction of cerebral capillaries (Hall et al., 2014).

Emphasis is given here to pericytes because they have been reported (Mughal et al., 2023) to wrap capillaries from arteriole to venule, in three variants (ensheathing, mesh, thin-strand). Capillary-wrapping pericytes have been described in the circulations of kidney, heart, muscle, lung and skin, where they appear to be fewer in number than in the cerebral circulation (Murray et al., 2017) and have yet to be shown to exert a level of control of blood flow comparable to that seen in the brain, mediated by the constriction of capillaries by the contraction of pericytes, and their dilation by the relaxation of pericytes. The control of capillary blood flow by brain activity seems distinct in extent; for this reason, we include capillary autoregulation in the “shared but specialised” list of protections (Table 1).

### Protection against neuronal depletion: adult neurogenesis

Neurogenesis is the generation, through cell division and differentiation, of neurones and neuroglia. In development, it is obviously essential to the formation of the brain. In adults, it seems an ideal response to counter losses of neurones that may occur during ageing. Adult neurogenesis is now established in animals, most clearly in rodents studied in laboratories [reviewed in Stangl and Thuret (2009)] and, within the brain, most clearly in the hippocampus, an area of cerebral cortex important for memory, the element of cognition lost first in the course of age-related dementia. Whether significant neurogenesis occurs in the adult human remains debated (Duque et al., 2022), but the recent development of protein makers of neurogenesis in the adult human brain (Moreno-Jimenez et al., 2021; Terreros-Roncal et al., 2023) has given investigators increasing confidence. The data seem clearest for one part of the cerebral cortex (hippocampal cortex) and, within the hippocampus, for one gyrus, the dentate, and indicate that neurogenesis persists in the dentate gyrus into late adult life, slowing with age and dementia. Conversely, however, Moreno-Jimenez et al. (2021), using single-cell transcriptomic analysis of hippocampal neurones, could find evidence of neurogenesis in several non-primate mammals, but not in humans.

The excitement arising from the possibility of neurogenesis in the adult human is that of mitigating age-related loss of cognition/dementia by upregulating adult neurogenesis. The issue is currently unresolved, for humans. In rodents, however, once the evidence seems clear, there followed a decade or more of testing whether neurogenesis could be upregulated, whether the age-related decline in the vigour of neurogenesis could be halted, and whether such interventions could mitigate, even reverse, age-related loss of cognition. The results have been impressive, in non-human species. In 2009, Stangl and Thuret (2009) reviewed studies published in the preceding decade and more. Their survey (summarised in their Tables 1, 2) showed evidence that neurogenesis can be accelerated by resilience-inducing stresses such as caloric restriction and dietary plant toxins and is impaired by vitamin deficiencies and high-fat diets; and that cognitive performance was improved or impaired by the same factors.

Finally, on this form of protection, there is one complication that might arise from neurogenesis in the adult brain. One reason that it was long taught that neurogenesis does not occur in adult humans is that there was little clinical evidence for it. For example, cancers of neurones—neuromas, which do occur, tragically, in children—are rare in the adult. Most cancers arise in tissues that constantly create new cells—the skin or the lining of the gut or the blood or the testis—as the result of mutations that dysregulate mitosis; no mitosis, no cancer. So, one unwelcome complication of upregulating neurogenesis in the ageing brain might be cancers of neurones. Such neuromas have yet to be described, and people with a diagnosis of dementia might well choose to take that risk. Before that choice arises, however, the reality of neurogenesis in the adult human brain needs to be established.

### Protection from the toxicity of extravasated blood

Biologists, by convention and logic, do not criticise evolution. Evolution has happened; criticism is irrelevant. If, however, one were allowed—just once—to criticise something in the evolution of vertebrates, the issue most deserving of criticism might well be the neurotoxicity of blood. Stokum et al. (2021), writing in the clinical context of stroke, argued that “…*interventions for CNS haemorrhage should be guided by the principle that blood is exquisitely toxic to the brain*.” They suggested that, although blood contains several neurotoxic components (their Table 2), most of the toxicity arises from haemoglobin (their Table 1). Other investigators (e.g., Kumar and Bandyopadhyay, 2005; Robinson et al., 2009; Dutt et al., 2022) have attributed the neurotoxicity of haemoglobin to the haem moiety, after it is released from its globin (becoming “free haem”) or to haemin, the oxidised form of haem; and sometimes to the iron released from haem (Fe^2+/3+^). In the last decade, investigators of iron homeostasis (Ma et al., 2022; Pan et al., 2022; Wu et al., 2023) have proposed an Fe-specific form of cell death—ferroptosis—and have suggested that it accounts for at least some of the cell death occurring in Alzheimer’s dementia (Ma et al., 2022; Wu et al., 2023). The suggestion was made without referring to the capillary haemorrhage thought by many to be a critical component of the pathogenesis of the dementia [reviewed in Stone et al. (2015)]. The attribution of the neurotoxicity of blood to free haem/hemin/Fe seems logical since the globin proteins are not known to be toxic. But Stokum et al. (2021) note that the separation of haem from haemoglobin takes place in identifiable steps and “nearly every stage of haemoglobin degradation is toxic.”

In evolutionary terms, haemoglobin and other haem proteins have evolved to serve important functions in vertebrates, the best known of which is the transport of oxygen in the blood. So, and this is the criticism, evolution has created a situation in which over 100 g of haemoglobin—highly toxic to brain tissue [and to other body tissues (Gbotosho et al., 2020)]—flows through the brain every minute, delivering the oxygen critical for brain function. What could possibly go wrong? Well, nothing goes wrong for decade after decade, as long as the haem is bound to its globin protein, and the haemoglobin is contained in red cells and the red cells within blood vessels. When the wall of a cerebral vessel bursts—and bleeds are increasingly understood as the core pathology of several age-related dementias (de la Torre, 2004; Cullen et al., 2006; Stone, 2008, 2015)—the red cells spill out of their vessel and their membranes break down, spilling haemoglobin into the neuropil. The mechanisms that clear blood after an intracerebral haemorrhage have been studied extensively. It has been reported, for example [reviewed in Fu et al. (2023)], that tissue-resident microglia and monocyte-derived phagocytes are the active cellular scavengers important for clearance. Before clearance is complete, however, two things happen: some of the haem binds to the neuropil, where it can be detected post-mortem with the Perls Fe^3+^ reaction (Cullen et al., 2005); and neurones die, presumably from the combined effects of (1) the hypoxia that results from vessel failure; (2) the excitotoxicity of glutamate in the blood (above) and (3) the toxicity of haemoglobin and its breakdown products. This toxicity, this evidence suggests, makes even small bleeds a driver—some argue the driver—of the pathology and cognitive loss of several dementias (Cullen et al., 2005; Stone, 2008; Stone et al., 2015; Johnstone et al., 2023). It just seems a bad idea—but then it was never an idea, it was evolution—to evolve a molecule as toxic as haemoglobin to deliver oxygen to the brain.

Intracerebral haemorrhage is then an explosion-like disruption of the stable-to-that-point structure of that bit of the brain, and the brain’s response to such explosions is beginning to be understood. In addition to the “debris clearance” just mentioned, two responses related to the toxicity of haem have been described. One response is the secretion by neural cells, induced by the hypoxia resulting from the vessel failure, of the proteins haemopexin, haptoglobin and haemoxygenase-1, all of which are considered to detoxify haem and contribute to its breakdown (Robicsek et al., 2020; Choi and Kim, 2022). Haemopexin levels, for example, are raised in the “Alzheimer” brain, particularly at the sites of plaques (Philbert et al., 2021); these authors suggest that in “Alzheimer’s” there is a “a widespread underlying microvasculopathy,” supporting the view (Cullen et al., 2006; Stone, 2008; Stone et al., 2015; Philbert et al., 2021) that plaques form at the sites of small haemorrhages from small vessels.

The second response is the hypoxia-induced secretion, also by neural cells, of a peptide known as Aβ, notorious as the alleged toxin driving Alzheimer’s dementia, an allegation of which the molecule is probably innocent (Cullen et al., 2006; Stone, 2008; Castellani et al., 2009; Stone et al., 2015; Brothers et al., 2018; Atwood and Perry, 2023). Reports going back three decades have described the physiological/trophic roles of Aβ [reviewed in Bishop and Robinson (2004), Castellani et al. (2009), Morley and Farr (2012) and Brothers et al. (2018)]. These tissue-positive roles of Aβ now include the facilitation of synapse formation and of the growth of dendrites (Puzzo et al., 2008, 2011; Puzzo and Arancio, 2013; Palmeri et al., 2017), the protection the brain from infections (Brothers et al., 2018) and the detoxification of haem (Chuang et al., 2012). Given the view, persisting in parallel, that Aβ is the toxin causing the neurodegeneration of age-related dementia (Alzheimer’s), some investigators have sought ways to resolve the trophic/toxic discrepancy. Morley and Farr (2012) and Puzzo et al. (2012) have argued, for example, in terms of hormesis—that Aβ is trophic at low doses, toxic at high. Others argued that the toxicity of Aβ arises not from its monomeric forms but from its oligomers or from the complex it forms when bound with free haem (Atamna and Boyle, 2006; Neumann et al., 2014; Roy et al., 2020; Nath et al., 2022). Some, as long ago as two decades (Bishop and Robinson, 2002; Robinson and Bishop, 2002), suggested that the discrepancy might not be resolvable—that Aβ may not be the toxic driver of Alzheimer’s dementia.

This issue is beyond the present scope (but watch this space). As part of its resolution, it would be natural to look for an alternate culprit, and there is one or more at hand: a toxin with form—the “exquisite neurotoxicity of haemoglobin” (Stokum et al., 2021) and the products into which it breaks down [see also Kumar and Bandyopadhyay (2005) and Robinson et al. (2009)]; the excitotoxicity of glutamate (above) and the hypoxia caused by the loss of blood supply to the area of tissue supplied by the haemorrhaging vessel.

### Protection against damage that occurs nevertheless: plasticity and re-wiring

There is considerable evidence that, when damage occurs to one part of the cerebral cortex, the function it served can be taken over by another, usually adjacent part of the brain. The renewal of function is not always complete, but the effect can be considerable. As examples, most survivors of strokes regain some function after the event, given time. Further there are many reports [beginning with Katzman et al. (1988)] of senile plaques in considerable numbers in the brains of individuals who had no symptoms before death. Evidently, the brain repairs its circuitry and function around these small foci of damage (typically 0.1 mm diameter or less). The effect is that the brain can re-establish normal function and, if the lesions are small, can do so without symptoms or signs. More generally, the brain functions better than would be expected from the known extent of damage (Katzman et al., 1988; Stern et al., 2019).

Self-repair is a property of many complex organisms and many tissues. Further, self-repair has now been pioneered in materials science, mimicking evolution (Speck and Speck, 2019). But there is a further element to the brain’s ability to self-repair. In addition to generating new cells and regrowing processes of damaged cells, the brain seems able to re-establish the circuitry necessary for its most mysterious and complex function – cognition, and the sense of self. And that is unique to the brain.

Issues much discussed in this field of cerebral rewiring and plasticity include limits of degree and time: how much damage can the brain repair, and still preserve full cognition and the sense of self and personality? What are the mechanisms of “re-wiring”? Does it involve active synapse formation and, if so, what mechanisms are involved? And, does this ability continue unabated throughout life? And if not, what diminishes it? These questions are often framed in terms of “cognitive reserve” or “brain reserve.”

## A note on cognitive reserve

Cognitive reserve is a concept developed to denote the ability of the brain to perform despite damage (Katzman et al., 1988); or to re-establish normal function after damage; or to recover from a challenge strong enough to send its function into delirium or unconsciousness (head trauma or anaesthesia) (Stern et al., 2019). “Reserve” is sometimes used concerning the function of the liver [hepatic reserve (Frager and Schwartz, 2020)] kidney [renal reserve (Palsson and Waikar, 2018)]. Each organ either has available or can generate anew tissue capable of maintaining full function, despite partial damage. As already noted above, the mechanisms of cognitive reserve seem particularly intriguing because of the complexity of brain circuitry that must be restored after damage, and the complexity of the function restored (cognition, the sense of self, consciousness).

Two features of cognitive reserve are clear, however, though their mechanisms remain elusive. First, the reserve diminishes with age. The old recover more slowly from brain damage, such as occurs in stroke, less completely and with greater institutionalisation and mortality (Lui and Nguyen, 2018), and they fall into confusion or delirium more readily (Potter et al., 2006; Mattison, 2020). Second, there appears to be an absolute limit to cognitive reserve; it can be exhausted. One way of understanding dementia, although the question is still debated (Fong et al., 2015), is that cognitive reserve is depleted with age as damage accumulates in the brain, caused for example by the increasing intensity of the ageing pulse (Bateman, 2004; O’Rourke and Safar, 2005; Henry-Feugeas, 2009; Stone et al., 2015) or by head trauma (Andersson and Stone, 2023), or a combination of the two (Johnstone et al., 2023). The degree of stress (fever, medication, psychological stress) required to induce delirium reduces correspondingly with age, and recovery takes longer, until (in this argument) confusion and cognitive loss occur without apparent stress and become permanent, and recovery is not possible.

Much remains to be learnt about cognitive reserve. We mention it here because it allows us to rephrase the evolutionary point of the protections of the brain considered above. Arguably, these mechanisms of protection conserve cognitive reserve; and, once we understand them, some of these protections—particularly acquired resilience—empower us to take steps to preserve our personal cognitive reserve, so that cognition survives longer than the biblical 70 years, for as much as two or three decades (Andersson and Stone, 2023).

## Discussion and summary: what guidance from this evolution-oriented analysis?

The protections described are a guide to factors that threaten the brain sufficiently for protections to have evolved. They perhaps offer a background to guide the individual and the medical professional in the optimisation of brain health, the basis of cognition and personality.

### The psychology of understanding

Table 1 lists the protections of the brain reviewed here, which prevent damage from external trauma; from hypoglycaemia and hypoxia, from extremes of blood pressure and from tissue breakdown generally (acquired resilience). Further, there seems to have evolved in the brain a “cognitive reserve” of recruitable neurones, an ability to rewire circuitry to overcome damage that does occur, such as the damage caused by capillary bleeds, despite these protections (Johnstone et al., 2023).

How we use this knowledge would likely be dependent on our mood. In an adventurous mood, one might say—*My brain is well-protected, so I can go out there and do stuff*, even exhilarating but brain-threatening stuff like head-first tobogganing. Or, conscious of the acceleration of the onset of dementias caused by trauma to the head and brain (Johnstone et al., 2023), one might say*—I will go out and enjoy life, just not an extended career in the front row of a rugby scrum*. Or, in a providential mood, one might say—*I will be really proactive. I will eat the plant-toxin-rich diets, exercise in my youth but avoid knocks to the head, use photobiomodulation to make my tissues resilient, exercise in mid-age, enjoy regular saunas, keep my weight under control, exercise into old age, endure managed hunger as in a 5*/*2 diet, and then more exercise. Then my brain-life might be very long*—and, a cynic might say, it might seem even longer. These are personal choices; understanding the evolution of brain protection helps make them informed choices.

### Another approach—epidemiology

The Lancet Commission on Dementia (Livingston et al., 2020) analysed published randomised controlled trials (RCTs) of a range of hypotheses of the causes of dementia, without an evolutionary emphasis. Where an RCT indicated a lifestyle correlate of dementia, like hypertension or alcohol consumption, the Lancet authors dubbed the correlate a “modifiable risk factor” (MRF). The Commission’s analysis led to recommendations to minimise one’s risk of dementia, including controlling hypertension, not smoking, avoiding air pollution, wearing a hearing aid if needed, avoiding head trauma, limiting alcohol consumption, a good education, avoiding obesity, preventing or controlling diabetes, addressing disturbances of sleep, good nutrition and avoiding noise pollution. The reader will readily identify recommendations that arise from both sources (evolutionary and RCTs) and those that arise only from one or the other. It would be a small step to form a list encompassing them all. And, of course, other approaches may be fruitful, such as analysis of the genetic mutations that predispose to or protect us from dementia.

Knowledge of the protections that have evolved, and of empirically demonstrated MRFs, thus yields guidance for those who seek a long cognitive life: the point common to these different approaches is perhaps the need for respect for the one brain with which we are each endowed. The brain may be the most vulnerable of our organs; it is also the organ central to our consciousness of self. Protection by conscious choice leads us to avoid what endangers the brain and to do what adds to its protection. This seems a common-sense choice. The protections considered above act to extend its fully functional life to the biblical three score years and ten. Even among centenarians, a minority retain full cognition (Corrada et al., 2010); so there is a decade or several of cognitive health to be gained or foregone by our choice.

## Author contributions

JS: Conceptualization, Investigation, Writing – original draft, Writing – review & editing. JM: Conceptualization, Investigation, Writing – review & editing. DJ: Conceptualization, Investigation, Writing – review & editing. SR: Conceptualization, Investigation, Writing – review & editing.

## Footnotes

^1^Psalms 90:10, King James version: The days of our years are threescore years and ten; and if by reason of strength they be fourscore years, yet is their strength labour and sorrow.
